# Point-of-Interest Preference Model Using an Attention Mechanism in a Convolutional Neural Network

**DOI:** 10.3390/bioengineering10040495

**Published:** 2023-04-20

**Authors:** Abbas Bagherian Kasgari, Sadaf Safavi, Mohammadjavad Nouri, Jun Hou, Nazanin Tataei Sarshar, Ramin Ranjbarzadeh

**Affiliations:** 1 Faculty of Management and Accounting, Allameh Tabataba’i University, Tehran Q756+R4F, Iran; a.bagherian@atu.ac.ir; 2 Department of Computer Engineering, Mashhad Branch, Islamic Azad University, Mashhad 9G58+59Q, Iran; sf.safavi@gmail.com; 3 Faculty of Mathematics and Computer Science, Allameh Tabataba’i University, Tehran Q756+R4F, Iran; nouri_mohammadjavad@atu.ac.ir; 4 College of Artificial Intelligence, North China University of Science and Technology, Qinhuangdao 063009, China; houjun202301@163.com; 5 Department of Engineering, Islamic Azad University, Tehran North Branch, Tehran QF8F+3R2, Iran; ab.tataee@gmail.com; 6 ML-Labs, School of Computing, Dublin City University, D04 V1W8 Dublin, Ireland

**Keywords:** social networks, attention mechanism, deep learning, POI

## Abstract

In recent years, there has been a growing interest in developing next point-of-interest (POI) recommendation systems in both industry and academia. However, current POI recommendation strategies suffer from the lack of sufficient mixing of details of the features related to individual users and their corresponding contexts. To overcome this issue, we propose a deep learning model based on an attention mechanism in this study. The suggested technique employs an attention mechanism that focuses on the pattern’s friendship, which is responsible for concentrating on the relevant features related to individual users. To compute context-aware similarities among diverse users, our model employs six features of each user as inputs, including user ID, hour, month, day, minute, and second of visiting time, which explore the influences of both spatial and temporal features for the users. In addition, we incorporate geographical information into our attention mechanism by creating an eccentricity score. Specifically, we map the trajectory of each user to a shape, such as a circle, triangle, or rectangle, each of which has a different eccentricity value. This attention-based mechanism is evaluated on two widely used datasets, and experimental outcomes prove a noteworthy improvement of our model over the state-of-the-art strategies for POI recommendation.

## 1. Introduction

The emergence of localization services and extensive application of mobile smart devices have impressively amended user experience and improved personalized service quality [1,2]. To improve the user experience and satisfy users’ demands and requirements of different users, personalized recommendation strategies using geographical location to recommend some points of interest (POIs) are broadly employed. Generally, a POI recommendation system is able to improve the productivity of organizations and is more complicated than the classic movie and news recommendation systems [3,4,5]. A POI recommendation system can combine individualized places and different information sources that meet their preferences at a certain time and location for users. These POI systems are subclasses of information filtering systems and are capable of fully analyzing a user’s preference, trajectory, and location and exploring prospective demands for recommending the best POI to target users. POIs normally include shopping malls, supermarkets, bakeries, dessert shops, restaurants, and so on [4,6,7].

The growth of POI recommendation systems using location-based social networks can bring numerous benefits, comprising meeting consumer demands, improving user experience, and so on. Real-time and contextualization of socialization lead to providing plenty of information and further diversifying the source of information for users. However, present techniques have a number of weaknesses, including insufficiently analyzing and fusing user-related information (i.e., spatial–temporal factors, preference, and social relationship), leading to obtaining low accuracy in terms of the recommendation and unexploited potential interests of users [8,9,10,11].

In the recent decade, location-based social networks (LBSNs) such as Yelp, Foursquare, Brightkite, and Gowalla, have been more popular. This popularity is because of the development of smartphones, the infrastructure of Internet networks, and the access of most people to social networks [4,8,12].

**Figure 1:**
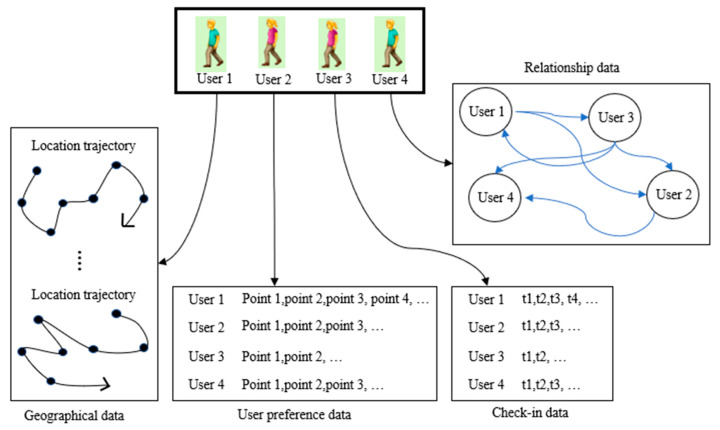
An example of a user-based recommended system.

These LBSNs obtain check-in information from each user, such as relationship data, information on the geographical of the visited locations (longitude and latitude), and user preference data. LBSNs additionally permit users to psychologically share their check-in interests, engage their audience and community, influence their emotional and visual behaviors and approaches, make new friends, and indicate user feelings [13,14,15]. Figure 1 demonstrates an example of LBSN, exhibiting the friendship between users and interactions between check-in activities. Point-of-interest (POI) recommendations can be used for personalizing the recommendation of places for mobile users in LBSNs and enable users to attain new locations in high-demand regions.

POI recommendation is a branch of the recommendation system, and its main purpose is to recommend unvisited or unseen places, i.e., restaurants, movie theatres, etc., to the users on the basis of their past history or even recommend the same preferred place on the basis of past best experience. POI recommendation systems use mostly place information and user social activities along with behavior to categorize the recommendation list of POIs [16,17].

Recently, employing deep learning (DL) and machine learning (ML) structures has been more popular in artificial intelligence (AI) applications, such as POI recommendation and computer vision [18,19,20,21,22,23]. DL models outline representation-learning strategies that are more trustworthy for learning hidden patterns inside the input data using various simple components or layers [4,15]. Each layer mines higher-level features from the prior layer. Consequently, various DL models can obtain different solutions to explore the intrinsic high-level features that are beneficial for recommendation tasks [24,25,26,27,28].

Yin et al. [29] proposed a DL model based on a spatial-aware hierarchical collaborative (SH-CDL) and heterogeneous features. Their model is capable of solving the problems of user preferences spatial dynamics using heterogeneous features and hierarchically additive representation learning and divergent attributes for spatial-aware user conclusions. A new POI recommendation strategy based on the mining the real-time data is designed in [30]. In order to learn inherent demonstration and explore textual information of POIs, they employed a CNN pipeline. Doan et al. [31] proposed a new approach for exploring user check-in on the basis of focusing on regional attraction and neighborhood competition.

In He et al. [32], a POI recommendation pipeline was implemented that considered many factors, such as social, time, and geographical factors. Their technique utilized the cascading combination and linear weighting. In order to combine a number of user’s features, a deep neural network (DNN) was designed by Ding et al. [33]. Their model learned the importance of user behavior in location-based social networks. In addition, the sparsity of data in POI recommendation systems was decreased using focusing on the effects of temporal, geographical, and categorical factors. Rahmani et al. [34] suggested a linear fusion of POI contexts based on a regression technique by combining the contexts for each (i) group of users or (ii) users from their historical relations. Wu et al. [35] proposed a distinguishing missed POI approach that focuses on visited locations by users at a previous certain time and builds a category-aware multigraph embedding model.

Liu et al. [36] presented a real-time point-of-interest (POI) recommendation model named RTPM. The model utilizes users’ check-in records and current times (without current locations) to generate POI recommendation lists based on the user’s current interests. RTPM mines the periodic trends of users’ patterns of life among weeks to model their long-term preferences. For short-term preferences, the model considers sequential influence as well as preferences with time restrictions, which is crucial for the real-time recommendation. Trainable time transition vectors are introduced to depict users’ current time preferences influenced by the public, while a category filter is used to remove POIs that users have little interest in. The model makes POI recommendations solely on the basis of users’ behavior patterns without using their personal attributes or current locations, thereby ensuring privacy protection.

Yu et al. [37] proposed a novel point-of-interest (POI) recommendation technique named NGPR, which addresses the challenge of recommending accurate and appropriate locations to users due to the sparsity of user check-in information. The proposed technique utilizes user-generated content, such as check-ins, and constructs a heterogeneous location-based social network (LBSN) graph of users, POIs, categories, and time periods. The Node2Vec technique is employed to establish the latent vectors of POIs and users, which captures the similarity of POIs and users on the basis of their neighborhood in the graph. Furthermore, the proposed method integrates comprehensive factors for POI recommendation, including category preference, POI popularity, and geographical distance. The category preference takes into account the user’s preference for specific types of POIs, while the geographical distance considers the user’s current location and the distance to recommended POIs. The POI popularity factor incorporates the popularity of the recommended POIs on the basis of the number of check-ins. Experimental results demonstrate that the proposed NGPR method outperforms state-of-the-art methods in terms of recommendation accuracy. The proposed method has practical applications in location-based services, such as travel planning and personalized marketing.

To address the above-mentioned issues and appropriately enhance the accuracy of a POI recommendation system, we applied an attention mechanism to the features of users and suggest a well-organized POI recommendation strategy that employs six input features, including user’s ID and check-in time (second, minute, hour, day, and month). Moreover, to improve the robustness and performance of the DL-based model, the geographical location data for each user is used for computing the eccentricity score. This score can be employed as an attention mechanism inside our CNN model and is multiplied by the weight of neurons before the fully connected layer.

The main contributions of this study are summarized as follows:

1. We suggest a two-route CNN model to extract key features more effectively.
2. This study attempts to address the issue in current POI recommendation techniques of unsatisfactorily merging a user’s relevant context, leading to unsatisfactory suggestions. Consequently, a well-organized POI recommendation model based on the user’s geographical position, check-in time, and preference is suggested.
3. An attention mechanism is used to investigate the effect of geographical position more accurately.

## 2. Material and Methods

The structure of the suggested POI model is represented as follows. The structure of our attention mechanism is described in Section 2.1. Our proposed CNN model is outlined in Section 2.2. Final outcomes and remarks are explained in Section 3. The conclusion is demonstrated in Section 4.

### 2.1. Attention Mechanism

A complex cognitive function inside the brain that is vital for human beings is called attention [38,39]. One key characterization of perception is that a human does not need to analyze all input information at once. As an alternative, humans are inclined to focus selectively on a part of the data where and when it is required but overlook other perceivable data at the same time. This technique can be considered to be a means for humans to select the most important information as soon as possible from massive amounts of data employing limited processing resources. This attention mechanism critically expands the accuracy and efficiency of perceptual information processing [40,41].

The attention mechanism is a powerful technique in machine learning and deep learning that has revolutionized the field of recommendation systems, particularly in point-of-interest (POI) recommendation systems. In a POI recommendation system, the attention mechanism can help to identify and highlight relevant POIs by selectively focusing on certain features of the input data. By focusing on the most relevant features, attention-based models can improve the accuracy and effectiveness of POI recommendations.

Accordingly, the attention mechanism can be utilized as a resource allocation structure that is used to explain the problem of data overload. So, when we are facing limited computing power, this method is able to process key features with limited computing resources. In addition, this technique can also be utilized to elucidate incomprehensible neural architecture behavior [41,42,43].

One of the key benefits of attention mechanisms for POI systems is the ability to incorporate a user’s preferences and past interactions into the recommendation process. For example, if a user has visited certain POIs in the past, an attention-based model can assign higher weights to those POIs and give them more importance when generating recommendations. This can help to personalize the recommendations and improve the user experience.

In addition, attention mechanisms can be used to incorporate spatial and temporal information into POI recommendations. For example, an attention-based model can assign higher weights to POIs that are located in the same neighborhood or are near each other in terms of distance. This can help to generate more coherent and relevant recommendations that are tailored to a user’s location and travel patterns.

Furthermore, attention mechanisms can be used to address the cold-start problem in POI recommendation systems, where new or infrequent users have limited or no data available for training. By incorporating external data sources, such as social network information, attention-based models can learn to generate recommendations, even for users with limited data.

Overall, attention mechanisms have become an essential tool for POI recommendation systems, enabling more accurate, personalized, and effective recommendations. Future research will likely focus on refining and improving these models further and exploring new ways to incorporate different types of data and information into the recommendation process.

Ranjbarzadeh et al. [43] suggested an attention mechanism to focus only on small parts of MRI images for brain tumor segmentation. Bahdanau et al. [44] proposed an attention mechanism to accomplish translation and alignment simultaneously on machine translation tasks.

**Figure 2:**
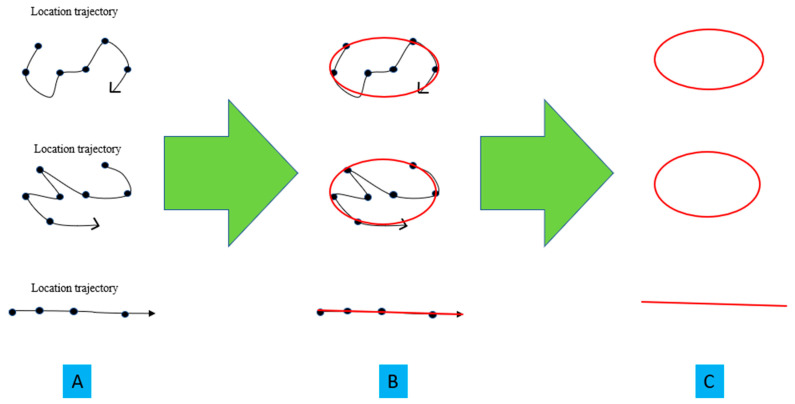
Some examples of finding a fitted shape for each location trajectory. (**A**) Location trajectory for three users, (**B**) trying to find the best shape that its border completely fitted with the trajectory, and (**C**) a fitted shape for each user’s trajectory.

In this paper, we use the geographical information map to create an eccentricity score as the attention mechanism. In other words, the trajectory of each user draws a shape, such as a circle, triangle, or rectangular, and each of them has a different eccentricity value. The eccentricity value indicates the ovalness of an ellipse, and the value near one outlines a high degree of ovalness. The eccentricity value of an ellipse is calculated by the ratio of its distance from the focus and the distance from the directrix. Some examples of the eccentricity ratio include (1) circle = 0, (2) line = infinity, (3) ellipse = between 1 and 0, and (4) parabola = 1. It means that if geographical data related to a user are similar to a line, its eccentricity rate is infinity [45,46,47]. Figure 2 indicates some examples of geographical data related to different users.

### 2.2. Proposed CNN Model

The growing opportunities for location-based social networks (LBSNs) come hand in hand with various difficulties, comprising greater-than-before data volumes and computational loads for analyzing data as well as further dissimilar data structures with cumulative dimensions (relationship data, geographical information of the visited locations (longitude and latitude), and user preference data) often featuring compound relationships. In addition, the LBSNs-related applications and tasks fields can differ significantly in their inherent requirements and processes [4,15,17].

In the context of data mining and analysis, deep learning models (DLs) are currently paving new avenues for POI recommendation systems. Unlike the earlier shallow neural network approaches, such as artificial neural networks (ANNs) that have been under exploration for many years, DL-based structures are characterized using a considerably amplified number of continuously linked neural layers [48,49,50,51]. This amplified number of layers is able to mine hidden patterns and higher-level features and can discover more difficult and hierarchical relationships. Simultaneously, growing the number of feature-extracting layers leads to deeper levels of complexity that commonly require more computational loads and training samples [52,53,54]. However, DL pipelines became very prevalent due to numerous practical developments, including cloud computing, high-performance graphic cards, and well-organized data processing strategies (e.g., pooling methods, non-linear activation functions, or data augmentation). These developments allow a model to efficiently calculate numerous non-linear transformations of the respective input samples (the ability of end-to-end learning) [23,26,55,56].

**Figure 3:**
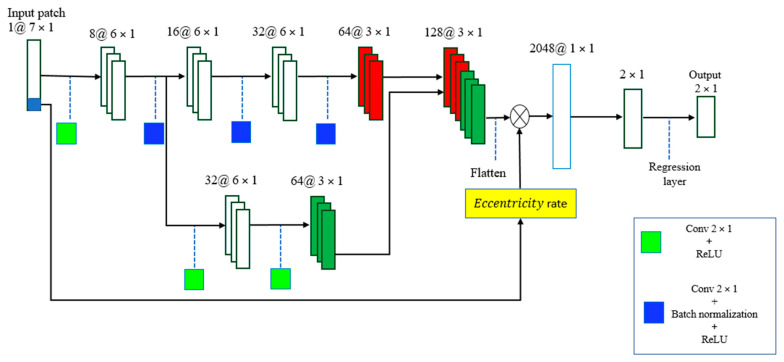
Our CNN attention-based pipeline.

Convolutional neural networks (CNNs) are subsets of deep learning models that are able to extract critical and informative hidden patterns from the input data and analyze this information, leading to better performance and robustness of the models. So, in this work, we utilized a CNN attention-based pipeline that used all visited sites, patterns of commute trips, and related time for each user [57,58]. This employed strategy uses 6 input feature maps comprising the user’s ID, day, second, minute, hour, and month of visiting time by each user for applying to convolutional layers and geographical data for applying to the attention mechanism. As is clearly shown in Figure 3, we used two main routes for using input features. In the first route, six out of seven input features are employed and the next route only accepts one feature (geographical feature). The first route is divided into two paths after the first convolutional layer to explore more critical features from input data.

Three convolutional layers (Conv layers) in the first path and two convolutional layers in the second path with a different number of kernels were used inside the model. We employed 8@6 × 1 kernels at the beginning to produce 8 feature maps. This size of filters (6 × 1) with large receptive fields is utilized in both feature-extracting routes. Moreover, these kernels with large receptive fields are helpful for extracting low-level features inside the input data. In addition, in each route, the size of kernels was reduced to 3 × 1 for extracting high-level features. After extracting low-level and high-level features, these features are concatenated to be ready to be flattened in the next step. The ReLU function and batch normalization layer are utilized in the first route to improve training and keep the output values within the normal range. In addition, in order to reduce the effect of overfitting the model and control the fitting process, one dropout layer with a 0.05% dropout probability is used before the regression layer to independently train the neurons. Before the fully connected (FC) layer, we apply the obtained attention value to all neurons for increasing the performance of our model.

Lastly, the regression layer is used to generate two values for specifying the anticipated location of the recommended locations on the basis of the learning dataset. The learning rate is 0.001, and the training process has 70 epochs.

Our CNN pipeline utilized the *RMSE* technique [59] to compute the discrepancy between true and predicted locations for minimizing the loss in Equation (1):

(1) $$RMSE=\sqrt{\frac{1}{n}\overset{n}{\underset{i=1}{\sum }}{\left(predicted\;locations-True\;locations\right)}^{2}}$$

where n implies the number of estimated locations.

## 3. Experiments

### 3.1. Datasets

In recent years, different spatial techniques were applied to real social networking datasets (Yelp and Gowalla). These datasets involve many geographical check-in behavior and information represented by longitude and latitude (See Table 1 for more information) [3,17]. Yelp is a well-known merchant review website and social networking operation that was founded in San Francisco in 2004. This website includes merchants in tourism, cinemas, hotels, shopping centers, restaurants, and other fields, with access by more than 184 million people by 2020. The Yelp dataset gives us access to 30,887 users, 265,533 social relations, 860,888 reviews, and 18,995 POIs [11,15]. Gowalla is the first mobile application and has users’ check-in information from February 2009 to October 2010. The Gowalla dataset entails 1,278,274 reviews, 18,737 users, 86,985 social relations, and 32,510 POIs. This dataset allows people to check into locations, create future plans, and discover new hot spots and permits customers to share experiences about what they see, find, and hear with relatives and friends [15,16].

### 3.2. Evaluation Measures

We assess the accuracy and performance of the suggested model and the baseline techniques utilizing two common evaluation metrics, namely Recall at N (Recall@N), and Precision at N (Precision@N) [1,3,49,57,60]. These two scores are computed by matching each estimated location outcome with its corresponding true locations for achieving the correct order of top-K POIs for a user. Recall implies the probability that the positive locations of the original locations are finally correctly predicted as positive locations. So, the higher value for recall means a better suggestion result. Precision implies the probability of appropriately guessing positive locations among locations anticipated as positive locations. So, the higher value for precision means a better recommendation result [25,61].

These criteria can be computed as follows [34,62]:

(2) $$\left\{\begin{array}{l} Recall@K=\frac{1}{\left|P\right|}\times \overset{U}{\underset{j=1}{\sum }}\frac{\left|TopK\left(U_{j}\right)\cap L_{j}\right|}{\left|L_{j}\right|},\\ Precision@K=\frac{1}{\left|P\right|}\times \overset{U}{\underset{i=j}{\sum }}\frac{\left|TopK\left(P_{j}\right)\cap L_{j}\right|}{\left|TopK\left(U_{j}\right)\right|}, \end{array}\right.$$

where $TopK\left(U_{j}\right)$ describes the peak value of K in the test samples for the proposed POIs, $L_{j}$ defines the POIs that the jth client has realized in the training information, and *K* stands for the different range (5 to 50) of recommended POIs for investigating the effectiveness of pipelines. Recall@K and Precision@K represent the fraction of realized POIs by the target client that is rewardingly proposed and a fraction from top-K recommended POIs to the expected user, respectively [15,24,25].

### 3.3. Experimental Results

Our suggested pipeline was run on a computer equipped with GTX 1060, 8 GB RAM, and Core i7 using Python (PyTorch library). We compared our strategy with the traditional and latest POI recommendation models to validate the performance of the proposed model.

- UFC [63]: This model attempts to incorporate check-in correlation, friend importance, and user preference for addressing problems of the sparsity of the user-POI matrix. Using collaboration filtering, the user favorite is individualized. As distant and close friends share a familiar influence, this model focuses on merging these two diverse factors to formulize friend importance.
- DeePOF [64]: This pipeline investigated the impact of the most analogous pattern of fellowship rather than using the pattern of the fellowship of all users. In order to detect the similarity, the mean-shift clustering strategy was employed. In addition, the most common friends’ spatial and temporal features were used to apply to a suggested CNN model. The output of some suggested convolutional layers can predict the longitude, latitude, and ID of subsequent right spots.
- HGMAP [3]: A multi-head attention mechanism inside a hybrid graph convolutional network with (HGMAP) was suggested to construct a spatial graph on the basis of the geographical gap between leverage graph convolutional networks (GCNs) and pairs of POIs for expressing the high-order connectivity among POIs. The HGMAP model was also used to address the difficulties in traditional recommender systems, such as data sparsity and cold start.
- LORE [65]: This model investigated the sequential influence on users’ check-in activities in LBSNs. The LORE strategy tries to explore sequential forms from a check-in position stream of all users. In addition, a tenth-order additive Markov chain (AMC) was used for computing the probability of a new location using user visiting information. The LORE model not only used the most recently visited locations for predicting a new visiting location but also employed the sequential influence more comprehensively.
- LFBCA [66]: In the LFBCA pipeline, the impact of social relations for each user was explored to suggest POIs. In addition, for characterizing the check-in behaviour, positions and users are added to the diagram. This model can handle the sparsity problem by incorporating the attributes of POIs into the recommendation process. However, it can still suffer from the cold-start problem, where there is not enough information available about a new user.
- APOIR [1]: This strategy is one of the primary POI recommendation systems based on an adversarial learning technique. The APOIR model has two main modules, including a discriminator and a recommender. These modules are able to be trained in a mutual manner for learning user inclination by considering both social relations and geographical. This model can handle the sparsity problem by incorporating the aspects of POIs into the recommendation process. However, it may require a large amount of data to learn the different aspects of POIs accurately.
- PG-PRE [67]: This technique proposes a new ensemble learning framework for point-of-interest (POI) recommendation in location-based social networks (LBSNs), named preference–geographical point-of-interest recommendation ensemble (PG-PRE). Traditional POI recommendation pipelines rely on discovering similar users and exploring their check-in histories to generate suggestions. However, such suggestions may be biased and lack variety. To overcome this limitation, PG-PRE constructs multiple similar user groups for a target user employing a roulette selection-based sampling technique to enhance the diversity of these groups. Each group generates a POI recommendation suggestion, and a Gaussian mixture-based technique is used to estimate the voting weight of each group. Lastly, a recommendation list for the target user is obtained by comprehensively considering the proposals of each group according to their corresponding voting weight.
- FG-CF [68]: This model suggests a novel approach for point-of-interest (POI) recommendation in location-based social networks (LBSNs) called friends-aware graph collaborative filtering (FG-CF). Traditional POI recommendation techniques suffer from the sparsity of check-in data, which GCN can overcome by capturing the high-order connectivity of POIs and users. However, social ties are ignored in most current graph-based approaches, which are crucial for POI recommendation in real-world scenarios. FG-CF incorporates social information into a user-POI graph, updating user embedding according to a user-POI correlation matrix. Interaction messages are constructed by integrating nodes’ ego embeddings, neighbor embeddings, and social embeddings.

The aim of this study is to analyze and gain an accurate sequence of top-K POIs for each user. The performance of seven structures is investigated over both datasets Yelp and Gowalla with respect to the number K from the suggested POIs by the utmost offer value. We demonstrate the performance of POI systems only when k ∈ {5, 10, 15, 20, 25, 30, 35, 40, 45, 50}, since a larger value of k is commonly ignored. 

**Figure 4:**
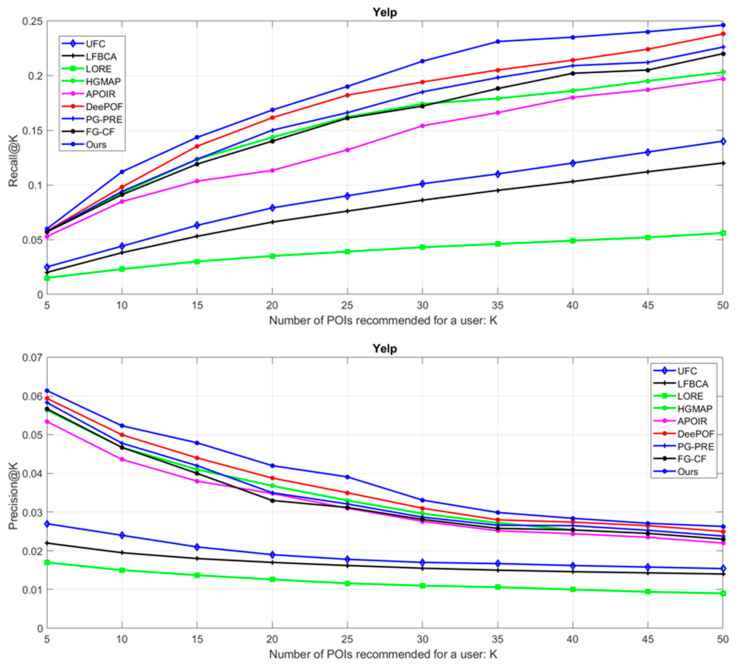
Comparison between our pipeline and six other structures for the Yelp dataset.

**Figure 5:**
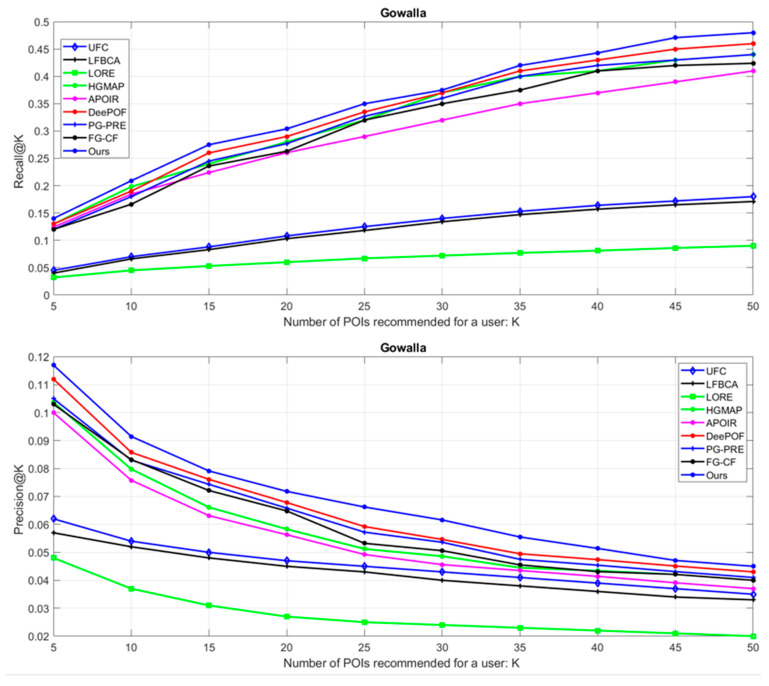
Comparison between our pipeline and six other structures for the Gowalla dataset.

The output results of the suggested strategy on Gowalla and Yelp datasets are informed in Figure 4 and Figure 5. As is shown, the suggested structure unfailingly outperforms all baselines on both datasets in terms of Precision@K and Recall@K. For example, in the Yelp dataset, our model obtains 0.062, 0.052, and 0.048 in terms of Precision@5, Precision@10, and Precision@15, respectively. In other words, our model indicates 0.04, 0.03, 0.002, 0.005, and 0.01 improvement of the performance for Precision@5 compared with FG-CF, PG-PRE, DeePOF, HGMAP, and APOIR, respectively. This benefit is rooted in the fact that the suggested two-route CNN is able to mine more critical features inside the input samples. Moreover, adding an attention mechanism improves the ability of the model to recommend the preferred locations for each user more accurately, especially for k < 30. Interestingly, as illustrated in Figure 4 and Figure 5, when K is not small, our pipeline is still talented at achieving better results. Although DeePOF, FG-CF, PG-PRE, APOIR, and HGMAP models could not accomplish better than the proposed strategy, they outperformed LFBCA, LORE, and UFC consistently in all criteria for assessment. Moreover, LORE and LFBCA approaches obtained the worst outcomes in all assessment criteria in both datasets. To further investigate the obtained outcomes, a paired *t*-test was employed for comparing the differences between the suggested model and the other pipelines, and it proved that the improvement of the suggested technique is statistically significant on both datasets (*p*-value < 0.01).

## 4. Discussion

The present paper discusses a novel approach to recommend points of interest (POIs) to users based on their geographical information. The approach uses a convolutional neural network (CNN) attention-based pipeline with an eccentricity score as the attention mechanism. The paper compares the proposed approach’s results to several other models, using precision and recall criteria for both Yelp and Gowalla datasets.

The authors introduce a new two-route CNN model for POI recommendation that employs different kernel sizes for convolutional layers. Unlike the deep structure typically used for CNN, the authors used a parallel pipeline to extract critical patterns inside the input samples. The model comprises three convolutional layers in the first path and two convolutional layers in the second path, each with a different number of kernels.

The authors further introduce an attention mechanism that focuses on the pattern of geographical information for each user in order to improve POI recommendations. They used six input features, including the user’s ID, day, minute, second, hour, and month of visiting time, and geographical data for applying to the attention mechanism.

The CNN attention-based pipeline technique utilized in this study is a type of DL pipeline that has been widely employed in computer vision tasks, such as object detection and image recognition. This approach involves a series of convolutional layers that extract relevant features from the input data, followed by pooling layers that downsample the features and reduce the dimensionality of the data. The output of the pooling layers is then fed into fully connected layers that perform classification or regression tasks. The attention mechanism used in this study focuses on specific regions of the input data that are more relevant for the recommendation task based on an eccentricity score that measures the distance between the user’s location and the POI.

The authors chose to use a two-route CNN model for POI recommendation, with different kernel sizes for convolutional layers. This approach allows the model to extract different features at different scales, which can be useful for capturing both local and global patterns in the input data. The parallel pipeline used in this study involves two separate routes, each with a different number of convolutional layers and kernels, that are combined at the end of the pipeline to create the final output.

The authors employed two popular datasets, Yelp and Gowalla, to assess the performance of the proposed approach as well as several other baseline models. They used precision and recall metrics to evaluate the accuracy of the recommendation system, which measures how many of the recommended POIs are actually relevant to the user’s interests and how many relevant POIs are missed by the system.

As indicated in Figure 4 and Figure 5, the precision and recall criteria measure the fraction of relevant results among the total results. A higher precision score indicates that the method retrieved a larger proportion of relevant results. The precision and recall values obtained for each model are given in Figure 4 and Figure 5 for different values of k, which is the number of recommendations provided to the user.

Comparing the precision results, it can be observed that the proposed model outperforms all other models for both Yelp and Gowalla datasets, achieving the highest precision values for all k values. As demonstrated in Figure 4, the precision of our model ranges from 0.0614 (for k = 5) to 0.0263 (for k = 50). The closest competitor to our model is DeePOF, which has precision values ranging from 0.0594 (for k = 5) to 0.025 (for k = 50). The other models, including UFC, LFBCA, LORE, HGMAP, and APOIR, have lower precision values compared with our model and DeePOF, with their precision values ranging from 0.027 (for UFC at k = 5) to 0.009 (for LORE at k = 50).

Comparing the results of the Yelp dataset using the recall criterion, we can observe that our model outperforms all other models for all values of k. At k = 5, our model achieves a recall score of 0.0598, which is the highest among all models. Similarly, at k = 50, our model achieves a recall score of 0.246, which is also the highest among all models. For lower values of k (5–15), the differences in recall scores among the models are relatively small. However, as k increases, the performance of our model becomes significantly better compared with the other models. For example, at k = 50, our model’s recall score is approximately 0.01 higher than the second-best model (DeePOF). The difference in performance is more pronounced for higher values of k, indicating that our model is better at recommending a larger number of relevant items.

As demonstrated in Figure 5, DeePOF and PG-PRE are the next best-performing models, with precision scores of 0.112 and 0.107, respectively, for k = 5 and decreasing scores for larger k values. LORE and LFBCA have lower precision scores than the other models, with LORE having precision scores ranging from 0.048 to 0.020 and LFBCA having scores ranging from 0.057 to 0.033. It is worth noting that a larger value of k is commonly ignored, as demonstrated by the decreasing precision scores for all models as k increases. Overall, our model appears to have the best precision performance for the Gowalla dataset.

From Figure 5, it is clear that our model consistently outperforms the other six models across all values of k, indicating its effectiveness in making POI recommendations. Specifically, your model achieves the highest recall values for all values of k. It can be observed that our model, DeePOF, and APOIR have similar patterns of recall values across different values of k. They all have a consistently increasing trend, with our model having the highest recall values at all values of k. In addition, none of the models shows a sudden decrease in recall values across different values of k, and all of them have a relatively smooth trend.

There are some significant differences in recall values between the models at higher values of k. For example, our model has a recall value of 0.471 at k = 50, while the second-best model (DeePOF) has a recall value of 0.46. Similarly, the recall values for APOIR are much lower than those of our model and DeePOF, with a maximum value of 0.41 at k = 50.

In addition, we can compare the performance of the models by considering the overall trend of their recall values as k increases. A good recommendation system should ideally maintain or improve its performance as k increases, indicating that it can provide good recommendations even when more items are recommended.

From Figure 4 and Figure 5, it can be observed that your model shows a consistently increasing trend in recall as k increases, indicating its ability to provide good recommendations even when more items are recommended. The other models, however, show more variability in their performance as k increases, indicating that they may be less effective at recommending a larger number of items.

## 5. Conclusions and Outlook

In this study, we suggest a new two-route CNN model for POI recommendation, which entails different kernel sizes for convolutional layers. Instead of using a deep structure for CNN, we employed a parallel pipeline for extracting critical patterns inside the input samples. Three convolutional layers (Conv layers) in the first path and two convolutional layers in the second path with different numbers of kernels were used inside the model. Moreover, an attention mechanism was introduced that focuses on the pattern of geographical information for each user. Furthermore, we used six input features, including user’s ID, day, second, minute, hour, and month of visiting time by each user for applying to convolutional layers and geographical data for applying to the attention mechanism. The attention mechanism and CNN pipelines used in the model address the issue of insufficiently analyzing and fusing user-related information in current POI recommendation techniques. The experimental outcomes based on two social networking dataset datasets (Yelp and Gowalla) indicate that our pipeline outperforms the state-of-the-art baselines.

However, the study has some limitations that should be addressed in future research. Firstly, the proposed model considers only six features of each user as inputs. While these features are important, there may be other relevant features that could improve the accuracy of POI recommendations. Future studies could consider incorporating additional features, such as user preferences or social relationships.

Secondly, the study evaluates the proposed model on only two datasets. While the results demonstrate the effectiveness of the model, it would be beneficial to evaluate the model’s performance on additional datasets to assess its generalizability and robustness.

Finally, the study’s training process has 70 epochs, which may be computationally expensive for some applications. Future studies could investigate ways to reduce the computational cost of the proposed model without sacrificing performance.

## Figures and Tables

**Table 1:** Data description for Gowalla and Yelp datasets.

	POIs	Sparsity (%)	Records	Social Relations	Users
Yelp	18,995	99.860	265,533	860,888	30,887
Gowalla	32,510	99.865	86,985	1,278,274	18,737

## Data Availability

The Yelp and Gowalla datasets are public datasets.
